# Growth Performance Is Driven by Site Conditions and Moderated by Functional Trait Plasticity in *Quercus robur* and *Prunus avium*

**DOI:** 10.1002/ece3.72978

**Published:** 2026-01-30

**Authors:** Alessandro Di Fabio, Samuel Aspalter, Debojyoti Chakraborty, Marcela van Loo, Lisa M. Rolke, Silvio Schüler, Robin Thiel, Christiane Veit, Jürgen Kreyling

**Affiliations:** ^1^ Institute of Botany and Landscape Ecology, University of Greifswald Greifswald Germany; ^2^ Department for Forest Growth, Silviculture & Genetics Austrian Research Centre for Forests Vienna Austria

**Keywords:** functional traits, growth performance, phenotypic plasticity, provenance trial, specific leaf area, spring phenology

## Abstract

Functional traits mediate plant‐environment interactions, yet their plasticity and genetic variability remain poorly quantified in long‐lived tree species. We examined provenance trial (common garden) data from one growing season of *Quercus robur L.* and *Prunus avium L.* across six sites spanning a ~2°C climate gradient to evaluate phenotypic plasticity and genetic differentiation in specific leaf area (SLA) and spring leaf‐out, and their effects on growth. We applied mixed‐effects models to separate provenance, family, and site effects, and to test trait‐growth relationships under contrasting water availability and temperature conditions. Growth performance from planting to between 16 and 8 years of age, respectively, was primarily determined by site conditions (modeled as a trial random effect, which includes climate, soil, and other local factors), with SLA emerging as the most significant functional trait across both species. As for the functional traits themselves, *P. avium* showed modest provenance‐level variation in both traits (*R* ^2^ ~ 7%), while in *Q. robur* only leaf‐out varied (*R* ^2^ ~ 19%) across provenances and families, indicating in both cases the presence of genetic differentiation. Plasticity in SLA and leaf‐out was detectable in response to climate variables but was generally small relative to inter‐individual variability (plasticity explained < 10% of trait variance). In *P. avium* , however, leaf‐out timing was strongly climate‐sensitive, consistent with its role as a short‐lived, pioneer species, whereas *Q. robur* (a long‐lived, dominant species) showed weaker plastic responses but slightly higher genetic structuring of traits. For all traits studied, a large proportion of the observed trait differences cannot be explained by climate or genetics, indicating high levels of individual variability (*R* ^2^ ~ 70%). This high inter‐individual variability, together with the modest but significant plasticity observed, suggests that the studied species possess the genetic potential (aided by plasticity) needed for acclimation and adaptation to climate change.

## Graphical abstract

Growth performance in *Quercus robur* and *Prunus avium* is strongly influenced by site conditions, but also by functional traits (Specific Leaf Area, spring leaf‐out date), which show moderate plasticity in response to climate. These traits differ among provenances of the same species, and there is variability in trait values within the provenances. The observed plasticity and inter‐individual variability suggest potential for acclimation and adaptation to climate change.

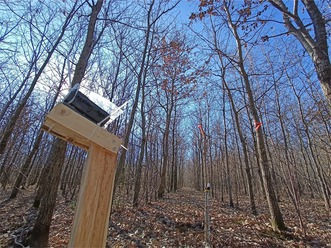

## 1 Introduction

Functional traits are “the characteristics of an organism that are considered relevant to its response to the environment and/or its effects on ecosystem functioning”, and they are a key determinant of the ecological role of a species (Dıáz and Cabido 2001). They are subject to the opposing evolutionary pressures of selection/local adaptation and genetic drift/gene flow (Savolainen et al. 2007). In general, species are not perfectly adapted to their local environment: most species present a wide range of variation in their functional traits, both due to individual genetic variability (Howe et al. 2003) and due to phenotypic plasticity of their genotypes (Alpert and Simms 2002).

Phenotypic plasticity (hereafter also “plasticity”) can be defined as “the range of phenotypes a single genotype can express as a function of its environment” (Nicotra et al. 2010). Plasticity has an important role in ensuring species fitness (Bradshaw 1965; Scheiner 1993). This is especially true for sessile, long‐lived species such as trees, which cannot migrate or put in place behavioral adaptations to overcome environmental challenges (Benito Garzón et al. 2019). In fact, due to its importance for survival, plasticity itself is subject to contrasting evolutionary pressures (Schneider 2022) balancing its limits (Valladares et al. 2007), costs (Van Buskirk and Steiner 2009), and benefits (Sultan 1995). Climate has been shown to be a driver of phenotypic plasticity across species (Stotz et al. 2021; Di Fabio et al. 2024) and populations (Ramírez‐Valiente et al. 2025). The exact amount of plasticity expressed for a given trait depends strongly on the evolutionary history and ecology of a given species or population. It is therefore very difficult to estimate a priori in the absence of experimental data (Nicotra et al. 2010). It is predicted that climate change will bring stronger and more frequent extreme weather events, and will lead to long‐term shifts in climate away from the climate optima to which trees have adapted in their evolutionary history (Valladares et al. 2014). Both effects will presumably favor individuals that are able to flexibly adapt to varying climatic conditions (Alpert and Simms 2002). Therefore, it is expected that plasticity will become increasingly important to ensure trees' survival in the face of climate change (Vázquez et al. 2017; Leites and Garzón 2023).

Two key functional traits in plant ecology are Specific Leaf Area (SLA) and spring phenology. SLA is defined as the ratio between leaf area and leaf weight and is a measure closely related to leaf thickness. High SLA is advantageous in moist climates, as it maximizes absorbed light while minimizing leaf production costs (Niinemets 2001) and has been associated with short‐lived, fast‐growing species due to the lower investment required (Reich et al. 1997). While differences in SLA across different species and biomes have been amply investigated (Wright et al. 2004), variations in SLA due to genetic differentiation among populations of the same species have also been shown in the literature (Stojnić et al. 2022). It is therefore natural to ask whether such differences might also be due to plasticity and not only local adaptation (Scheepens et al. 2010; Ramírez‐Valiente et al. 2010). Spring phenology is highly relevant for the life‐cycle of temperate and boreal plants, as proper synchronization with climate conditions and other species is necessary to ensure their survival and reproduction (Chuine and Beaubien 2001). Accordingly, at least for some tree species, it has been shown to be subject to evolutionary trade‐offs, for example, between risk of late‐frost damage and the opportunity to exploit more days of growth (Gömöry and Paule 2011). Deciduous trees regulate their exit from dormancy based on photoperiod length but also on other environmental cues such as temperature (Bradley et al. 1999; Vitasse et al. 2010; Malyshev et al. 2022). This makes spring phenology potentially sensitive to climate change (Parmesan and Yohe 2003), including the possibility of phenological mismatch between trees and their environment (Linderholm 2006; Richardson et al. 2013).

Building on the evidence collected, we aimed to answer the question of how strong the effect of SLA and spring phenology on tree growth is, and how plastic these two functional traits are to climate conditions. Therefore, we investigated provenance trials (also known as common gardens) of two important European broadleaf tree species, finding in both cases that functional traits are significantly correlated with growth rates from planting to between 16 and 8 years of age, respectively, and that climate conditions significantly affect functional traits.

## 2 Materials and Methods

### 2.1 Study Species

*Prunus avium* L. (hereafter also “cherry”) is a fast‐growing, pioneer species, which prefers well‐lighted environments and is capable of strong vegetative growth. It is a relatively short‐lived tree (70–100 years), which is usually replaced by climax species, and is afterward found in disturbed patches or intermixed with more dominant tree species (Russell 2003). Cherry can adapt to different soil types, but it favors relatively light soils with good drainage, as it is sensitive to waterlogging. At the same time, it is vulnerable to drought, particularly in summer, due to its tendency to develop a shallow root system (Stojnić et al. 2022). Cherry is of high economic importance for humans, both for fruit and hardwood production, with its wood being one of the most valuable hardwoods produced in Europe (Welk et al. 2016). In addition to its economic value, cherry is an important component of forest ecosystems, being a food source for insects, birds (Breitbach et al. 2012), and mammals (López‐Bao and González‐Varo 2011). However, the ecosystem services it provides are at risk of being strongly impacted by climate change, in particular due to its high drought sensitivity and vulnerability to short‐term waterlogging (Wiström et al. 2023; Pérez‐Girón et al. 2024).

*Quercus robur* L. (hereafter also “oak”), together with its closely related congeneric *Q. petraea* , is “among the economically and ecologically most important deciduous forest tree species in Europe” (Ducousso and Bordacs 2003). It is extremely long‐lived and can grow to great sizes, with individuals often reaching 30 m of height. Currently it is cultivated mainly for its durable heartwood, which is a valued material in construction and high‐quality furniture manufacturing (Ducousso and Bordacs 2003). Oak is a moderately drought tolerant species, thanks to its relatively thick leaves and its deep taproot, which often allows it to access groundwater in dry periods (Eaton et al. 2016). Due to its high adaptability to a wide range of climate conditions and soil characteristics, it is a very widespread species, whose dispersal is additionally aided by mammals and birds. Similarly to cherry, oak requires high light levels, especially for its establishment, and can act as a pioneer species thanks to the large seeds it produces. It tends not to form pure stands, as it is often out‐competed by more shade tolerant species such as beech ( *F. sylvatica* ), except in wetter sites such as lowlands, where it can survive its competitors thanks to its tolerance to flooding, or in warmer and drier sites, where it is aided by its drought tolerance (Eaton et al. 2016). Some studies predict that the drier (and more variable) conditions brought on by climate change will increase oak dominance in European forests (Scharnweber et al. 2011; Mette et al. 2013). On the other hand, this effect might be counterbalanced by other biotic interactions, for example by increased insect activity (Sallé et al. 2014).

### 2.2 Trial Sites

**FIGURE 1:**
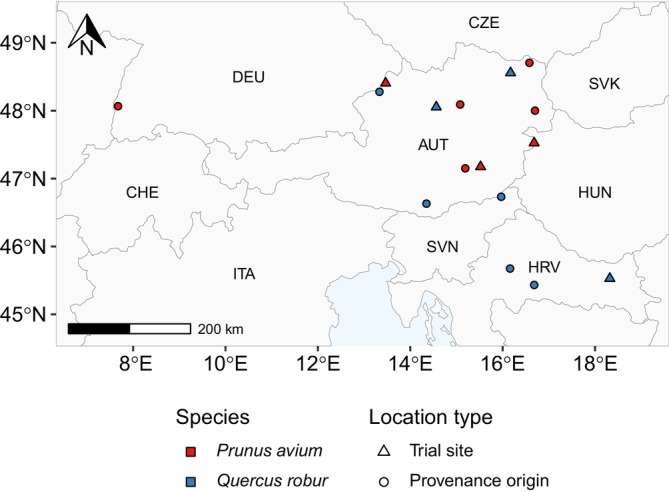
Map of the trial sites and provenances' origins, in Austria and neighboring countries. Red and blue points represent locations corresponding to *P. avium* and *Q. robur* , respectively. Triangles represent the location of the six trial sites, while circles represent the origin of the studied provenances. Country boundaries sourced from GADM database (Database of Global Administrative Areas 2022).

We worked on six different trial sites, three per species (Figure 1, Table 1). The three oak trials were established as part of the “PROEICHE” project (Schüler and Weißenbacher 2010), while the three cherry trials were established under the management of the Austrian Research Centre for Forests (BFW).

**TABLE 1:** Climate at trial sites. The climate data was sourced from Worldclim (Fick and Hijmans 2017), and is based on the 30‐year climatic normal for the period 1970 to 2000.

Country	Trial site	Latitude, °N	Longitude, °E	Elevation, m	MAP, L m^−2^	MAT, °C
*Prunus avium*						
AUT	Suben	48.40	13.47	391	901	8.4
AUT	Kumberg	47.17	15.52	530	863	8.6
AUT	Nikitsch	47.52	16.68	270	647	9.6
*Quercus robur*						
AUT	Weistrach	48.05	14.56	380	769	8.4
AUT	Weyerburg	48.56	16.17	312	565	9.2
HRV	Koška	45.53	18.32	100	675	10.9

Abbreviations: MAP, mean annual precipitation; MAT, mean annual temperature.

All three cherry trial sites, located in Austria, were established in 2009 using a randomized plot design with replicates. Each site contains 27 plots, with three replicates for each of the nine cherry provenances being tested. The arrangement of the plots was randomly determined within the trial site. Each plot consists of three columns of nine cherry trees, all originating from the same provenance. Alternating columns of oak ( *Q. robur* and *Q. rubra* ) and hornbeam ( *Carpinus betulus* ) were planted between the columns of cherry trees. The spacing between columns is two meters, while the spacing of trees within each column is 1 m.

Two of the oak trial sites are located in Austria, and beginning in 2006, the oak trees intended for these sites were grown from seeds and nurtured in the experimental garden of the BFW. In autumn 2007, the trees were planted in the Weistrach site. After a delay of several months due to the onset of winter, the Weyerburg site was established in spring 2008. The 1‐year‐old oak saplings were planted following a randomized plot design, with three block replicates. Each plot contains trees from a single provenance, spaced in a one by 2 m grid. The location of each plot within the blocks was randomly determined. Likewise, the location of the individuals within the plots was randomly determined. Both trial sites were surrounded by wildlife fences. More detailed information about the setup and subsequent management of the trial site can be found in Schüler and Weißenbacher (2010).

The third oak trial is located in Croatia. Oak seeds from the same collection campaign that provided the seeds for the Austrian trials were germinated and nurtured at a nursery operated by the Croatian Forest Research Institute from 2007 to 2010. After 3 years of growth, in spring 2010 the saplings were transplanted to the trial site. The experimental design is a randomized plot design with replicates, similar to the one described above for the Austrian oak trials. However, the trees were planted in a 2.5 × 2 m grid, and individuals from the same family were planted in the same row, instead of being randomly interspersed within each plot. All young plants were protected using Tuley tubes. More detailed information about the setup and subsequent management of the trial site can be found in Bogdan et al. (2017).

### 2.3 Growth Rate Measurement

Tree growth was measured at regular intervals by the managing institutions. Diameter at breast height and height of every living tree were measured approximately every 5 years, during winter, using a diameter measuring tape and a Vertex hypsometer (Haglof, Sweden), respectively. We used the most recent set of height measurements (2022 for all oak trials, 2019 for the Suben and Nikitsch cherry trials, 2017 for the Kumberg cherry trial) to calculate the average yearly height increment for each tree. In other words, we calculate the growth rate of each individual tree as *growth rate = height/age at measurement*. This linear approximation of growth rates is appropriate since the trees are relatively young (i.e., 16 years of age for the oaks, 8–10 years for the cherries), and therefore their growth has not yet slowed down due to senescence.

### 2.4 Provenances and Families

A total of 22 different provenances of oak and nine provenances of cherry were planted across the experimental sites. From this broad selection, we focused on a subset of five provenances per species (Figure 1, Table 2), aiming to capture a broad gradient of climate conditions, covering a wide range in both temperature and precipitation at their origins. The climate data used for this selection was sourced from the WorldClim database version 2.1 (Fick and Hijmans 2017), based on the 30‐year climatic normal for the period 1970–2000. We used historical climate data for provenance characterization because we aimed to quantify the climate conditions to which the provenances have presumably adapted in their population history.

**TABLE 2:** Climate at provenances' origin. The climate data was sourced from Worldclim (Fick and Hijmans 2017), and is based on the 30‐year climatic normal for the period 1970 to 2000. “ID” refers to the identification numbers used in Schüler and Weißenbacher (2010) and in the database of the BFW.

Country	ID	Provenance	Latitude, °N	Longitude, °E	Elevation, m	MAP, L m^−2^	MAT, °C
*Prunus avium*							
AUT	1	Königshof	48.70	16.58	343	605	9.1
AUT	2	Sommerein	48.00	16.70	163	590	9.9
AUT	4	Mayr‐Melnhof	47.15	15.19	771	983	7.6
AUT	5	Petzenkirchen	48.09	15.08	288	679	8.7
DEU	7	Liliental	48.07	7.67	322	1065	10.2
*Quercus robur*							
AUT	1	Geinberg	48.28	13.33	363	918	8.5
AUT	13	Halbenrain	46.73	15.97	233	825	9.5
AUT	14	Klagenfurt	46.63	14.35	439	930	8.2
HRV	18	Kutina	45.43	16.68	101	918	11.4
HRV	21	Turopoljski Lug	45.67	16.16	113	874	10.7

Abbreviations: MAP, mean annual precipitation; MAT, mean annual temperature.

For each oak provenance, 22 mother trees were selected at the time of seed collection, and the information on the identity of the mother tree that produced the seeds was recorded. This allows testing not only for differences at the provenance level, but additionally for differences among families of the same provenance.

### 2.5 Functional Traits

#### 2.5.1 Specific Leaf Area

At the end of August 2022, well after the end of the leaves' growth, we sampled six leaves, respectively, from around 50 trees at each trial site. We made sure to include the trees under observation by cameras (see next paragraph), and selected additional trees at random from the chosen provenances. The collection procedure mostly followed the guidelines outlined in Pérez‐Harguindeguy et al. (2013, chap 3.1). We ensured to consistently select leaves from the sun‐exposed upper part of the tree canopy, as sunlight exposure and position within the tree crown can influence leaf parameters (Pérez‐Harguindeguy et al. 2013, chap 3.1; Niinemets et al. 2015). After collection, leaves were placed immediately in a leaf press to preserve them throughout the fieldwork. The leaves were subsequently fully dried out by placing them in a drying cabinet for 24 h at 40°C and an additional 24 h at 70°C until constant weight. They were then kept completely dry by placing them in sealed containers with silica gel beads before being weighed. Three leaves per individual were employed for subsequent analysis, while three were reserved and put into storage in paper bags. The dry leaves were weighed using a milligram‐accurate scale, while their surface area was measured using the Leafscan App (Anderson and Rosas‐Anderson 2017) on an iPad (Apple Inc., Cupertino, USA). SLA was calculated as SLA = dry leaf area (cm^2^)/dry leaf weight (g). The SLA dataset was cleaned by removing five entries with obviously wrong weight measurements.

#### 2.5.2 Spring Phenology

**FIGURE 2:**
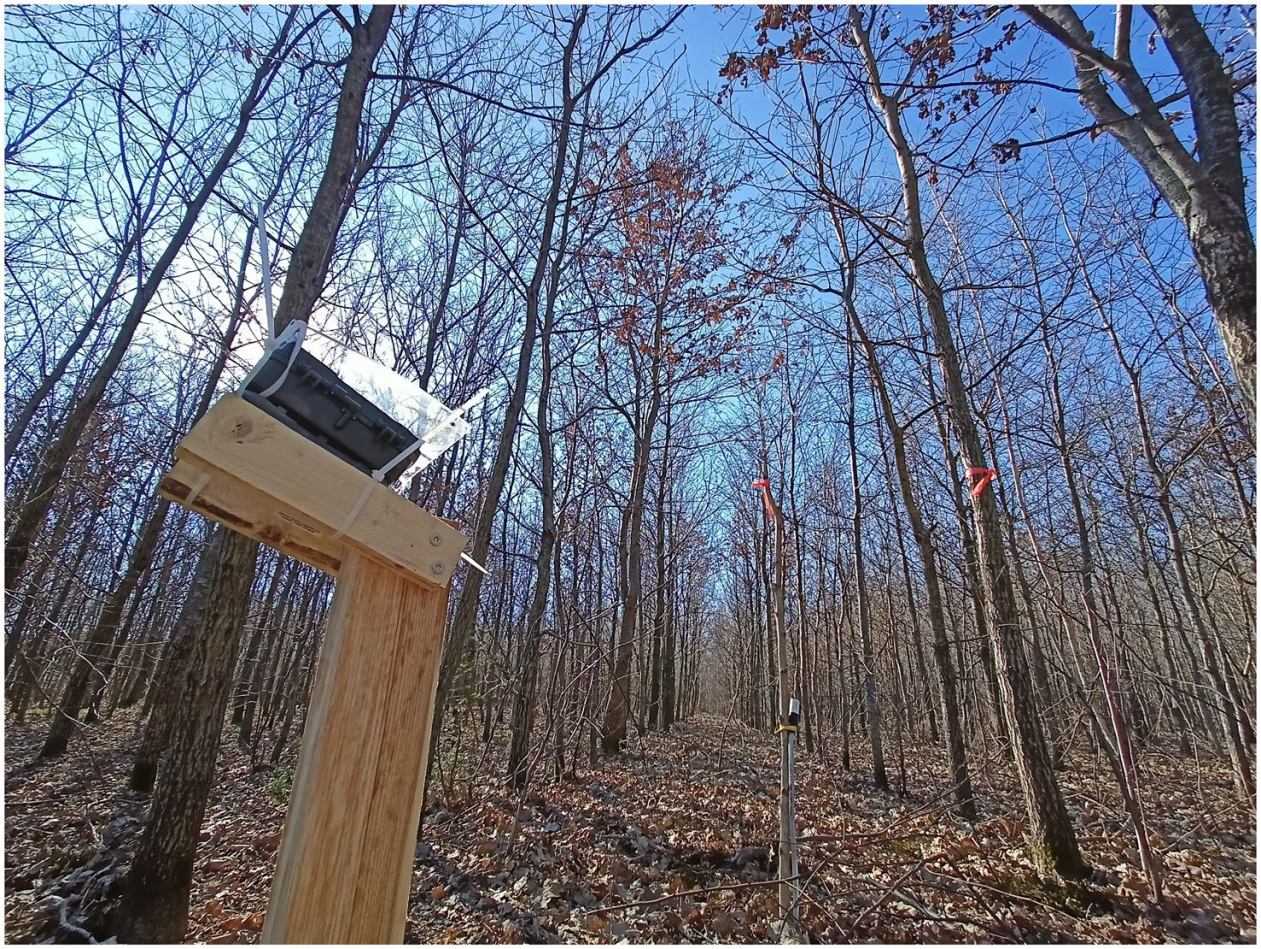
Example of a provenance trial: The Weyerburg site in late winter 2022. In the foreground is one of the cameras, mounted on a wooden pole. Further back is the above‐ground part of a soil sensor.

**FIGURE 3:**
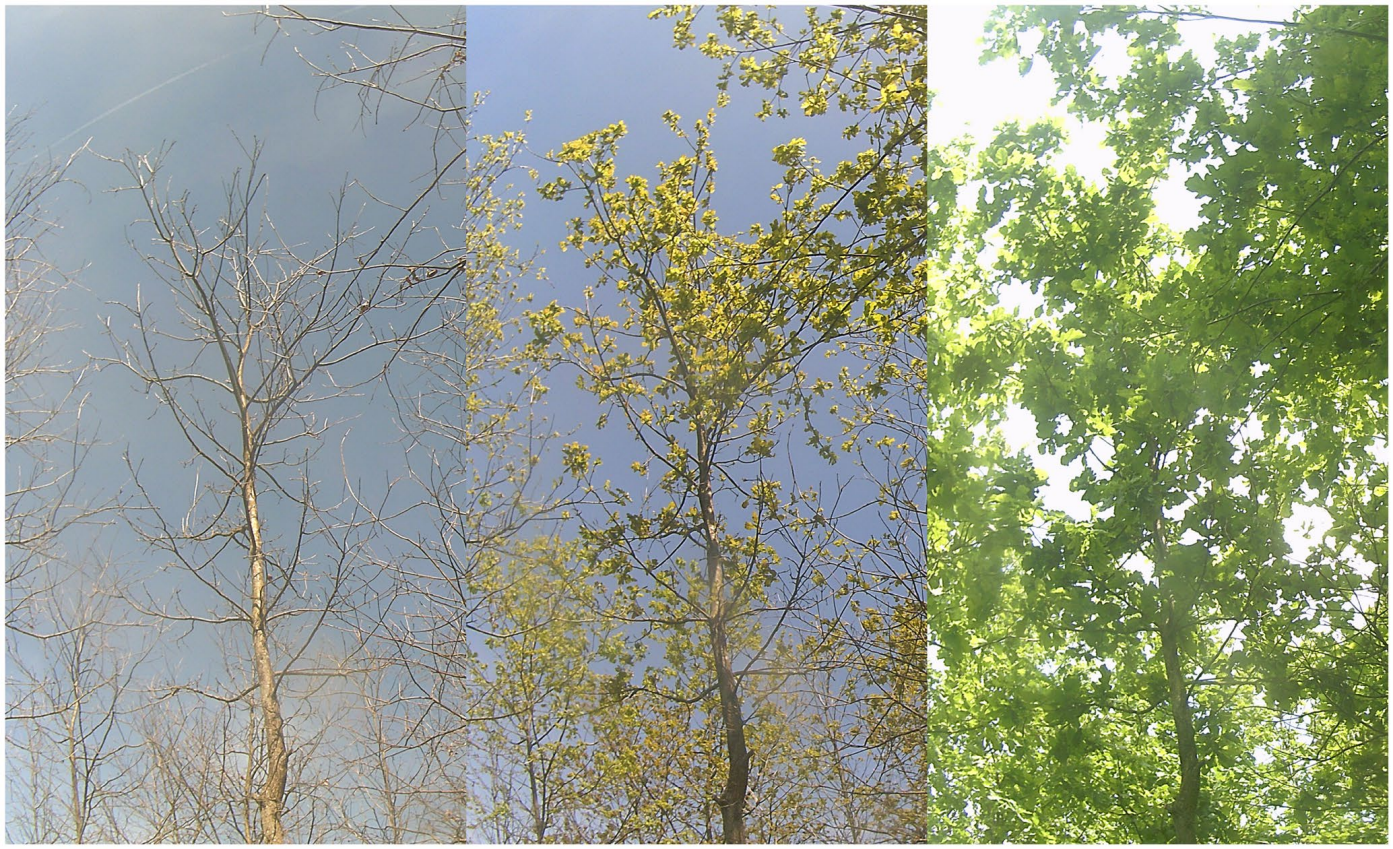
Cropped images from one of the installed cameras, exemplifying leaf development for a single tree. From left to right: Before leaf‐out; at the SoS date, when leaves are approximately 50% expanded; leaves completely expanded.

In March 2022, before the start of the growing season, we set up commercial wildlife camera traps (model 31,646, Berger + Schröter GmbH, Iserlohn, Germany; hereafter “cameras”) to take pictures of the tree canopy at regular intervals. The cameras (120 in total) were installed on the trunk of nearby trees or on wooden poles placed between the rows of trees and pointed upwards at the canopy of target and neighboring trees (see Figure 2 for an example). A transparent acrylic cover was additionally placed on the cameras to prevent rain from accumulating in the lens. The cameras were programmed to take a picture each day at 11:00, 12:00, and 13:00 to increase the probability of at least a picture per day being free of sun reflections or other obstructions. After collecting the data from the cameras in early 2023, the images were manually analyzed to visually identify the Start of Season date (SoS). We defined the SoS date (i.e., the date of transition from winter to spring) for each individual as the date where its leaves appear approximately 50% expanded (see Figure 3 for an example). We subsequently express SoS dates as days of deviation from the median date for each species. We chose to employ a manual evaluation approach, as automated image analysis methods based on color indices (such as Excess Greenness or Green Chromatic Coordinate) have shown to perform very poorly in previous trials with our experimental setup. This is likely due to the highly varying lighting conditions experienced by the cameras, as they are pointed skywards and not towards the ground. To prevent inconsistencies that might arise by variability in judgment between observers, all images were analyzed by a single person.

### 2.6 Climate Data

All climate sensors were installed during the course of the same fieldwork campaign, in March 2022.

Three weather stations (HOBO MX2305, Onset Computer, Bourne, USA) were installed at each trial site in the canopy of randomly selected trees. These stations measure relative air humidity and temperature every 15 min. We used these two variables to calculate the Vapor Pressure Deficit (VPD) at each measurement time. For each site, we calculated the daily 7‐day rolling mean of all sensor readings. Subsequently, we calculated the mean VPD over the relevant time interval for the two functional traits we studied. In particular, for the purpose of regressions against SoS, we aggregated the VPD from the date of sensor installation to the first SoS date of each site. For regressions against SLA, instead, we aggregated the VPD from sensor installation to 2 weeks after the first SoS date of each site, since we assume that leaf development might still respond to climate conditions for some time after leaf‐out, until the leaf is fully grown (Fender et al. 2011).

A similar setup was used for measuring soil climate parameters, with three TOMST buriable TMS units (Wild et al. (2019), TOMST s.r.o., Prague, Czech Republic) measuring soil temperature at three different depths (10 cm above‐ground, 10 and 50 cm below‐ground), in addition to relative water content at 50 cm below‐ground. The sensors were calibrated using soil samples collected at the trial sites and applying the calibration curves provided by the manufacturer. We applied the same per‐site 7‐day rolling mean and subsequent aggregation described for the air sensors in the preceding paragraph. For the purpose of all subsequent analyses, we employed only the temperature measurements taken 50 cm below‐ground, as they are less influenced by daily fluctuations due to sunlight and transient weather conditions.

### 2.7 Statistical Analysis

For each statistical analysis, we first set up a full linear mixed effect model including all relevant effects. Subsequently, we simplified the models in a stepwise selection process, aiming to balance model complexity and explanatory power (Murtaugh 2009). The first step was to ensure model convergence and non‐singularity of the parameters by simplifying the random slope terms. In case this was not sufficient, we removed in turn the random intercept term with the lowest Standard Deviation until model convergence. Next, we applied the step() function from the “lmerTest” package to further simplify the models, according to their Akaike Information Criterion (AIC). See Table 3 for details on the number of data points included in each model, the full models we started from, and the final models selected. All models were checked for significant deviations from statistical assumptions using the check_model() function from the “performance” package. We extracted the *R* ^2^ values from the fitted models using the r2() function from the “performance” package, which calculates marginal and conditional *R* ^2^ values following Nakagawa and Schielzeth (2013). The marginal *R* ^2^ takes into account only the fixed effects' variance, while “the contribution of random effects can be deduced by subtracting the marginal *R* ^2^ from the conditional *R* ^2^” (Lüdecke, Makowski, Ben‐Shachar, et al. 2025).

**TABLE 3:** Summary of the models employed, using “lme4” formula notation (Bolker 2024).

Species	*N*	Full formula	Final formula
*Growth rate ~ Functional traits*			
Cherry	55	growth_rate ~ sla*sos + (sla*sos|trial) + (sla*sos|provenance)	growth_rate ~ sla*sos + (1|trial)
Oak	105	growth_rate ~ sla*sos + (sla*sos|trial) + (sla*sos|provenance/mother_tree)	growth_rate ~ sla + (1|provenance) + (1|trial)
*SLA ~ Climate*			
Cherry	145	log(sla) ~ temp*moist*vpd + (temp*moist*vpd|trial) + (temp*moist*vpd|provenance)	log(sla) ~ moist + (1|provenance)
Oak	156	log(sla) ~ temp*moist*vpd + (temp*moist*vpd|trial) + (temp*moist*vpd|provenance/mother_tree)	log(sla) ~ temp
*SoS ~ Climate*			
Cherry	86	sos ~ temp*moist*vpd + (temp*moist*vpd|trial) + (temp*moist*vpd|provenance)	sos ~ temp + moist + (1 | provenance)
Oak	375	sos ~ temp*moist*vpd + (temp*moist*vpd|trial) + (temp*moist*vpd|provenance/mother_tree)	sos ~ temp + moist + (1|provenance/mother_tree)

Abbreviations: moist, soil volumetric water content; *N*, number of data points for the model; sla, specific leaf area; sos, start of season deviation; temp, soil temperature; vpd, vapor pressure deficit.

#### 2.7.1 Growth Rate ~ Functional Traits

We investigated the relationship between growth rate from planting to 16 years of age for oak and 8–10 years for cherry, and the functional trait values. The full model included an interaction term between SLA and SoS, and random slope and intercept terms for trial, provenance, and mother tree (only for oak). The final model for cherry included the interaction between SLA and SoS, and a random intercept for trial. The final model for oak included a term for SLA, and a random intercept for provenance and trial.

#### 2.7.2 SLA ~ Climate

We investigated the relationship between SLA and spring climate conditions, calculated from data measured on site as detailed in Section 2.6. We log‐transformed SLA, as it is a strictly positive real number, to improve normality of model residuals and model fitting. The full model included an interaction term between soil temperature, soil water content, and VPD, and random slope and intercept terms for trial, provenance, and mother tree (only for oak). The final model for cherry included a term for soil water content and a random intercept for provenance. The final model for oak included only a term for soil temperature.

#### 2.7.3 SoS ~ Climate

We investigated the relationship between SoS and spring climate conditions, calculated from data measured on site as detailed in Section 2.6. The full model included an interaction term between soil temperature, soil water content, and VPD, and random slope and intercept terms for trial, provenance, and mother tree (only for oak). The final model for cherry included terms for soil temperature and soil water content, and a random intercept for provenance. The final model for oak included terms for soil temperature and soil water content, and a random intercept for provenance and mother tree.

### 2.8 Software

We used R version 4.5.1 (2025‐06‐13) (R Core Team 2025) in RStudio version 2025.5.0.496 (Posit Team 2025) for all data preparation, statistical analysis, and figure making.

Data manipulation employed functions from the “tidyverse” (Wickham 2023) package collection, and from the “janitor” (Firke 2024) package. Geospatial data was manipulated using the packages “terra” (Hijmans 2025) and “tidyterra” (Hernangómez 2023). The “lme4” (Bates et al. 2025) and “lmertest” (Kuznetsova et al. 2017) packages were used for model fitting and selection. *R* ^2^ values were obtained and model assumptions were checked using functions from the “performance” (Lüdecke, Makowski, Ben‐Shachar, et al. 2025) package. Explained variance values were obtained using functions from the “insight” package (Lüdecke, Makowski, Patil, et al. 2025). Model predictions were obtained using functions from the “modelbased” (Makowski et al. 2025) package.

## 3 Results

### 3.1 Growth Rate ~ Functional Traits

**FIGURE 4:**
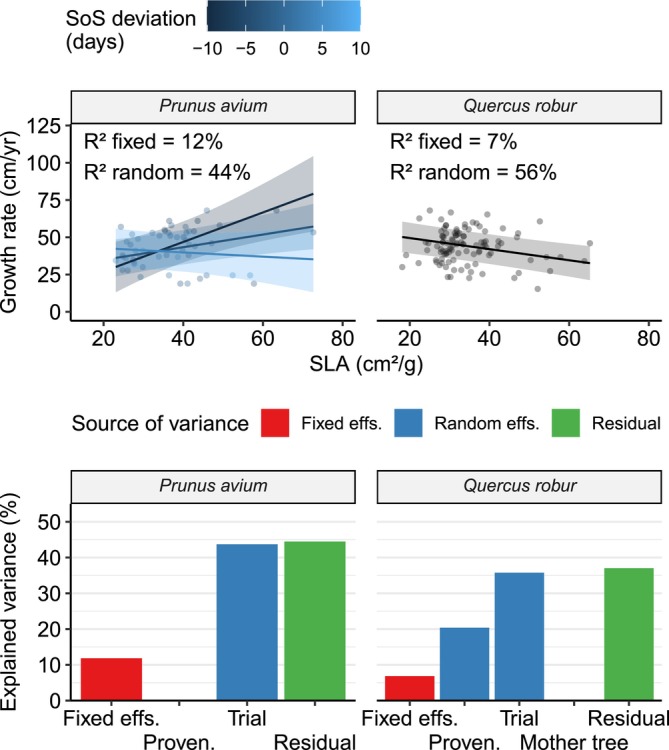
Top: Growth rate regressed against functional traits. Each point represents an individual tree. Regression lines for the fixed effects are overlaid on the data points. Shaded bands correspond to the 95% confidence interval for the regressions (these don't include random effects). Different colors represent the growth response to SLA at different values of SoS deviation (only relevant for *P. avium* ). Bottom: Variance partitioning of the corresponding models. Each bar represents the approximate percent variance explained by the relevant term(s) in the models. The “Fixed effs.” bar includes all fixed effects included in the respective model, as depicted in the upper scatterplot. The “Residual” bar refers to variance unexplained by the model. Missing bars correspond to terms which were discarded during model selection. SLA, Specific Leaf Area; SoS, Start of Season.

For both species, growth rate was correlated with the examined functional traits, and random effects seemed to have a bigger impact on growth rate compared to the examined fixed effects (Figure 4).

In cherry, we found an interaction between SLA and SoS deviation. For late‐flushing trees (SoS deviation = 10 days), SLA did not have a significant effect on the growth rate. On the other hand, median to early‐flushing cherry trees (SoS deviation = 0 and = −10 days, respectively) seemed to benefit from increased SLA: an increase of 1 cm^2^/g led to a predicted increase in growth rate of 0.42 ± 0.29 and 0.99 ± 0.61 cm/year, respectively. Trial site was the only significant random effect, explaining approximately 44% of the variance in the data. The fixed effects of SLA and SoS accounted for approximately 12% of the variance in the data.

In oak, an increase in SLA of 1 cm^2^/g led to a predicted decrease in growth rate of −0.37 ± 0.18 cm/year. In contrast, SoS deviation did not seem to impact the growth rate. Trial site explained approximately 36% of the variance in the data, while the provenance term explained 20%. The fixed effect of SLA accounted for approximately 7% of the variance in the data.

### 3.2 SLA ~ Climate

**FIGURE 5:**
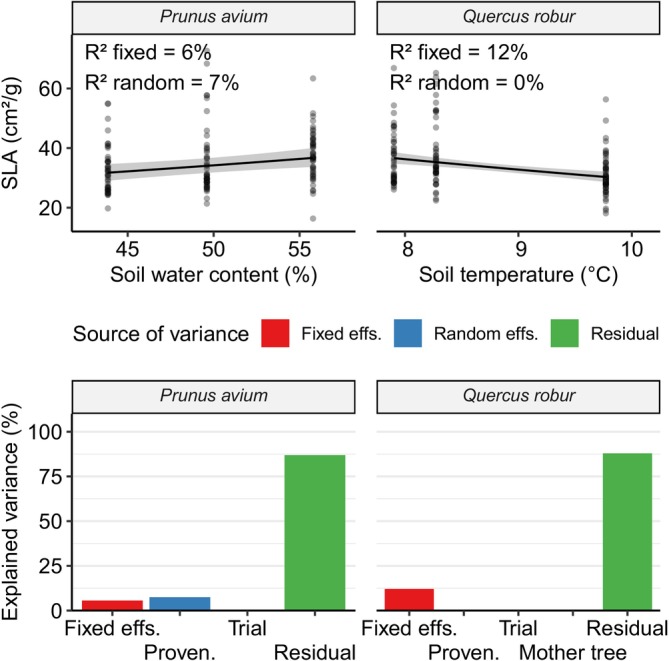
Top: SLA regressed against climate parameters. Each point represents an individual tree. Regression lines for the fixed effects are overlaid on the data points. Shaded bands correspond to the 95% confidence interval for the regressions (these don't include random effects). While the model has been fitted on log‐transformed SLA values, the figure shows the data on a linear scale. Bottom: Variance partitioning of the corresponding models. Each bar represents the approximate percent variance explained by the relevant term in the models. The “Fixed effs.” bar includes all fixed effects included in the respective model, as depicted in the upper scatterplot. The “Residual” bar refers to variance unexplained by the model. Missing bars correspond to terms which were discarded during model selection. SLA, Specific Leaf Area.

For both species, VPD did not seem to influence SLA values and was not included in the final models (Table 3). Overall SLA could be very weakly explained by the models as evidenced by the very high residual variance (Figure 5).

In cherry, SLA responded to soil water content. An increase of 10% led to an increase in SLA of 12% ± 8.4%. Provenance was the only significant random effect, explaining approximately 7% of the variance in the data. The fixed effect of soil water content accounted for approximately 6% of the variance in the data.

In oak, SLA responded to soil temperature, with an increase of 1°C leading to a decrease in SLA of −9.7% ± 3.9%. There were no significant random effects, while the fixed effect of soil temperature accounted for approximately 12% of the variance in the data.

Interestingly, SLA did not seem to be influenced by trial site effects in either species, and the effect of provenances' (and mother trees') genetics was small to non‐significant.

### 3.3 SoS ~ Climate

**FIGURE 6:**
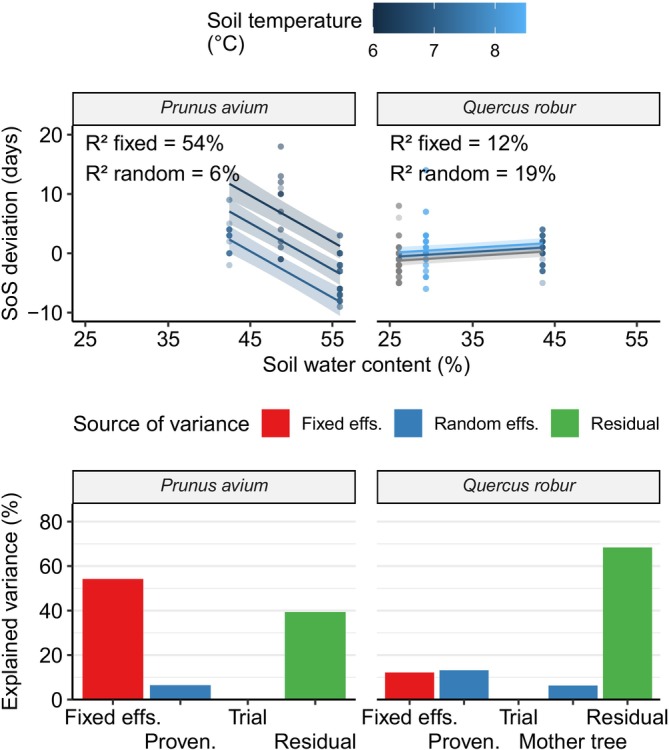
Top: SoS deviation regressed against climate parameters. Each point represents an individual tree. Regression lines for the fixed effects are overlaid on the data points. Shaded bands correspond to the 95% confidence interval for the regressions (these don't include random effects). Different colors represent the response of SoS to soil water content at different values of soil temperature. Bottom: Variance partitioning of the corresponding models. Each bar represents the approximate percent variance explained by the relevant term(s) in the models. The “Fixed effs.” bar includes all fixed effects included in the respective model, as depicted in the upper scatterplot. The “Residual” bar refers to variance unexplained by the model. Missing bars correspond to terms which were discarded during model selection. SoS, Start of Season.

For both species, VPD did not seem to influence SoS date, as SoS date depended only on soil water content and on soil temperature (Figure 6). However, this relationship differed in both direction and strength between the two species. In both species, SoS date could be partially explained by climatic and genetic effects, but the dependency on climate was much stronger for cherry, as evidenced by the much bigger variance explained by fixed effects (Figure 6).

In cherry, SoS deviation was well explained by soil water content and temperature (fixed effects *R* ^2^ = 0.54), with the only significant random effect being a small provenance effect (random effects *R* ^2^ = 0.06). An increase in soil water content of 10% led to a predicted earlier SoS by 7.8 ± 1.7 days. An increase in soil temperature of 1°C led to a predicted earlier SoS by 13 ± 3.5 days.

In oak, on the other hand, we found that SoS deviation was weakly explained by soil water content and temperature (fixed effects *R* ^2^ = 0.12). Furthermore, the effects of provenance and, interestingly, of the mother tree explained a bigger part of the variability in our data (total random effects *R* ^2^ = 0.19) compared to cherry and compared to the models describing SLA. An increase in 10% soil water content led to a predicted later SoS by 0.86 ± 0.31 days. An increase in soil temperature of 1°C led to a predicted later SoS by 0.65 ± 0.3 days. The provenance and mother tree terms explained respectively 13% and 6% of the variance in the data.

## 4 Discussion

### 4.1 Growth Rate Depends on Functional Traits

We found that, in both species studied, differences in functional traits can partially explain the trees' growth rate (Figure 4). To the best of our knowledge, the relationship between growth rates over multiple years and functional traits measured in a single year has not been investigated in the literature. Long‐term growth (from planting to 8–10 years of age for cherry or 16 years for oak) was correlated significantly with the traits measured in the study year. While year‐to‐year variation in trait expression exists, the correlation we found in the study year suggests that individual trees tend to express consistent phenotypes which translate into more or less growth. Oak trees with higher SLA values (thinner and larger leaves) show lower growth rates, probably due to higher transpiration losses at the relatively dry conditions of the trial sites. Interestingly, in cherry the relationship between SLA and growth rate is mediated by phenology: SLA doesn't significantly impact growth for late‐leafing trees, while early‐leafing trees show higher growth rates, but only if they also have higher SLA values. This may be due to cherry being a relatively drought sensitive species (Welk et al. 2016; Stojnić et al. 2022), combined with its ecological strategy as a pioneer species (Russell 2003). Our interpretation is that early‐leafing trees are able to exploit the higher photosynthetic potential and resource savings of thinner leaves, more than compensating for the potential losses incurred due to summer drought. In addition to climate effects, there seem to be genetic differences in growth rate among oak provenances, in agreement with previous results (Bogdan et al. 2017). However, this is not the case for cherry. The absence of a provenance effect on growth rate, in addition to growth depending on leaf traits, might mean that an individual's suitability to environmental conditions (e.g., an individual tendency to leaf out earlier) plays a bigger role than intrinsic growth rate differences among provenances. In both species we found a large effect of the trial site on growth rates. This is in agreement with the literature, which finds that site characteristics, such as soil composition, slope, and groundwater availability have a very strong impact on growth performance (e.g., Buras et al. 2020; Unterholzner et al. 2024). Still, a large proportion of variance in growth rates remains unexplained by the models, pointing to high levels of inter‐individual variability.

### 4.2 SLA Is Plastic, but Also Highly Individual

We found significant plasticity in SLA, for both species studied (Figure 5). In both species, the effect of climate on SLA seems to be an example of adaptive plasticity, as it follows the expectation that drier or warmer conditions would lead to lower SLA values (Poorter et al. 2009). On the other hand, while significant, these effects explain a small part of the variance in SLA. Additionally, SLA cannot be explained by effects of trial site, mother tree, or provenance (except for a very small provenance effect in cherry). The absence of a trial site effect indicates that site‐wide conditions, such as soil characteristics or climate effects not included in the models, don't significantly affect SLA. Likewise, there seems to be low genetic differentiation in SLA values among the provenances of the two species studied. These points, together with the high amount of variance unexplained by the models, suggest that, while there is a significant amount of plasticity in SLA in the two species, there is a large amount of individual variability that is not directly explainable by environmental or population‐level effects. This is in agreement with a recent review study on provenance variation in European trees (Aspalter et al. 2025), which found that variation among provenances and provenance‐environment interactions are less prevalent for leaf morphological traits than for growth rate. In some cases, studies report genetic differences among climatic groups, but not in general among provenances, as observed in a related *Quercus* species ( *Q. suber* , in Ramírez‐Valiente et al. 2010). The limited set of provenances in our study could also explain the lack of differentiation among them, which might become detectable if marginal provenances from the species' climatic limits were included. The high individual variability we found might be partially explained by microsite‐scale differences in sun and wind exposure (even at the leaf scale, Petruzzellis et al. 2017), or by genetic variability among individuals (Matesanz et al. 2020; Schmeddes et al. 2023), which has been shown to be very high at least for some traits and populations of oak (Caignard et al. 2018).

### 4.3 Spring Phenology Is Highly Plastic in Cherry, Not So in Oak

We found a very strong plastic response of spring phenology in cherry, and a very small one in oak (Figure 6). In both species, soil water content and soil temperature affect phenology, but the direction of the relationship is opposite, as warmer and moister conditions lead to earlier leaf‐out in cherry trees and later leaf‐out in oak. The difference in effect sizes we measured might reflect differences in ecological niches between the two species. For cherry, which often acts as a pioneer species or grows in gaps within the canopy of dominant tree species, it is advantageous to leaf‐out early, as soon as conditions are favorable, to exploit sunlight before other trees. However, while leafing out earlier might be advantageous in favorable conditions, it intrinsically carries risks due to climate unpredictability (e.g., late frost events). Oak, instead, is a longer‐lived species that often dominates in forest stands. For an ecologically dominant species, plasticity in spring phenology might be of smaller adaptive importance, as individuals would not need to exploit openings in the shade of other trees. A longer‐lived, dominant species might therefore profit from a less plastic, consistently later leaf‐out date as a less risky strategy. In both species studied, we did not find a significant trial site effect on spring phenology. We found instead significant but small effects of provenance (and mother tree in oak). This indicates a greater degree of genetic differentiation in spring phenology compared to SLA. The small size of the provenance and mother tree effects indicates, similarly to what we found for SLA, that individual differences in spring phenology due to genetics are present but smaller than differences due to individual variability.

### 4.4 VPD Doesn't Affect SLA nor Phenology

Interestingly, VPD does not significantly affect SLA nor spring phenology, contrary to the expectation that the amount of moisture in the air close to the leaves would affect their development. For SLA, it is likely that, since leaves are hydraulically connected to the rest of the tree, general water availability (better measured by soil water content) is the determinant factor in guiding leaf development, in addition to temperature (Fender et al. 2011). As for spring phenology, the absence of an effect of VPD is not surprising, as it has been shown that spring warming (in addition to winter chilling) is the main driver of leaf‐out in cherry and oak (Zohner and Renner 2014).

### 4.5 Uncertainties and Limitations

An important limitation of the present study is that we could test only a relatively small amount of species (two species, no conifers), provenances (five per species), environmental conditions (three per species), and individuals. We employed a model selection process to identify the variables to use in the final explanatory models. The removal of a variable from a model through model selection is not proof that variable has no biological significance, but a hint that its potential effect in the given data is small or absent. While we can be reasonably confident in the statistical significance of the relationships we did find, it is still possible for the non‐significant and removed variables to have an effect, yet one that we couldn't measure with the present data set. Testing more provenances and individuals, in a wider variety of climate and site conditions, would probably help in detecting such effects (and possibly their interactions). Another point is that, in the present study, we relate growth rates averaged over multiple years with functional traits measured in a single year. It would certainly be worthwhile to measure both growth rates and functional traits on a yearly basis, to see if plasticity is consistent year‐to‐year, and if it is adaptive in every year/climate. However, setting up a large‐scale network of provenance trial locations is a long‐term project, requiring international collaboration and consistent funding, both for managing the sites and for carrying out the measurements, making such experiments hard to establish.

## 5 Conclusion

The relative importance of phenotypic plasticity, local adaptation, and individual variability in the context of climate change has been amply discussed in the literature (e.g., Aitken et al. 2008; Jump et al. 2009; Nicotra et al. 2010). In the present study, we tested only two functional traits, and therefore cannot directly make predictions on the future of the species. However, we have consistently found significant plasticity and high individual variability across the species and parameters studied. The importance of individual variability in determining functional traits suggests that forest management strategies for climate change adaptation should focus not only on selecting the best‐adapted provenances but also on preserving the genetic diversity within populations. It seems plausible that high individual diversity, together with the significant plasticity we found, will give tree species the potential to adapt and acclimate to future conditions brought by climate change.

## Author Contributions

**Alessandro Di Fabio:** conceptualization (equal), data curation (equal), formal analysis (equal), investigation (equal), methodology (equal), software (equal), supervision (equal), validation (equal), visualization (equal), writing – original draft (equal), writing – review and editing (equal). **Samuel Aspalter:** investigation (equal), writing – review and editing (equal). **Debojyoti Chakraborty:** data curation (equal), writing – review and editing (equal). **Marcela van Loo:** data curation (equal), resources (equal), writing – review and editing (equal). **Lisa M. Rolke:** investigation (equal), writing – review and editing (equal). **Silvio Schüler:** funding acquisition (equal), project administration (equal), resources (equal), writing – review and editing (equal). **Robin Thiel:** investigation (equal), writing – review and editing (equal). **Christiane Veit:** investigation (equal), writing – review and editing (equal). **Jürgen Kreyling:** conceptualization (equal), funding acquisition (equal), project administration (equal), supervision (equal), writing – review and editing (equal).

## Funding

The authors declare that financial support was received for the research, authorship, and/or publication of this article. This research was funded by the German Federal Ministry of Food and Agriculture through the Fachagentur für Nachwachsende Rohstoffe within the framework “Waldklimafonds,” project EVA (FKZ2220WK08C4).

## Conflicts of Interest

The authors declare no conflicts of interest.

## Data Availability

The data and code that support the findings of this study are openly available in OSF at https://osf.io/u56p7/?view_only=a5c3fae9d5cc44a48a41c97b13d001a5 (read‐only link for peer review), reference number u56p7.
